# Supplementary material for: Development of a Multidisciplinary Aerodigestive Program: An Institutional Experience
Source: Children (Basel). 2021 Jun 23;8(7):535. doi: 10.3390/children8070535 (PMC8307318; doi:10.3390/children8070535)
Supplement: Supplementary file 1 [file children-08-00535-s001.zip › children-1271432-supplementary.pdf]

Supplemtary

Table S1. Questionnaire to the patient's caregiver.

**How do you think about your child's main aerodigestive symptom improvement after the aerodigestive program?**
**☐ Not improved at all ☐ Not improved ☐ Somewhat improved ☐ Improved ☐ Improved very much**
**Do you think the quality of life of your child has improved after participating in the aerodigestive program?**
☐ Not improved at all ☐ Not improved ☐ Somewhat improved ☐ Improved ☐ Improved very much
**How satisfied is your family with the aerodigestive program?**
☐ Unsatisfied at all ☐ Unsatisfied ☐ Somewhat satisfied ☐ Satisfied ☐ Satisfied very much
**Has the burden of the primary caregiver decreased?**
☐ Not decreased at all ☐ Not decreased ☐ Somewhat decreased ☐ Decreased ☐ Decreased very much

Table S2. Questionnaire to the ADT members.

**Did you know about the name or concept of ADT before participating in ADT?**
**☐ Did not know at all ☐ Knew roughly ☐ Knew concretely**
**Has the practice or treatment policy changed in your field before and after ADT?**
☐ Changed significantly ☐ Somewhat changed ☐ Not changed at all
**Did you increase the amount of time you spend on your patients before and after ADT?**
☐ Increased ☐ Not changed ☐ Decreased
**Do you think the patient's quality of care changed before and after ADT?**
☐ Improved ☐ Not changed ☐ Worsened
**Did your understanding of patients change before and after ADT?**
☐ Improved ☐ Not changed ☐ Worsened
**Has the degree of understanding of the work of medical staff in other fields changed before and after the ADT was implemented?**
☐ Improved ☐ Not changed ☐ Worsened

ADT, Aerodigestive team.
